# The relationship between body system-based chronic conditions and dental utilization for Medicaid-enrolled children: a retrospective cohort study

**DOI:** 10.1186/1472-6831-12-28

**Published:** 2012-08-07

**Authors:** Donald L Chi, Nicholas A Raklios

**Affiliations:** 1 Department of Oral Health Sciences, University of Washington School of Dentistry, Box 357475, Seattle, WA, 98195-7475, USA

## Abstract

**Background:**

Dental care is the most common unmet health care need for children with chronic conditions. However, anecdotal evidence suggests that not all children with chronic conditions encounter difficulties accessing dental care. The goals of this study are to evaluate dental care use for Medicaid-enrolled children with chronic conditions and to identify the subgroups of children with chronic conditions that are the least likely to use dental care services.

**Methods:**

This study focused on children with chronic conditions ages 3-14 enrolled in the Iowa Medicaid Program in 2005 and 2006. The independent variables were whether a child had each of the following 10 body system-based chronic conditions (no/yes): hematologic; cardiovascular; craniofacial; diabetes; endocrine; digestive; ear/nose/throat; respiratory; catastrophic neurological; or musculoskeletal. The primary outcome measure was use of any dental care in 2006. Secondary outcomes, also measured in 2006, were use of diagnostic dental care, preventive dental care, routine restorative dental care, and complex restorative dental care. We used Poisson regression models to estimate the relative risk (RR) associated with each of the five outcome measures across the 10 chronic conditions.

**Results:**

Across the 10 chronic condition subgroups, unadjusted dental utilization rates ranged from 44.3% (children with catastrophic neurological conditions) to 60.2% (children with musculoskeletal conditions). After adjusting for model covariates, children with catastrophic neurological conditions were significantly less likely to use most types of dental care (RR: 0.48 to 0.73). When there were differences, children with endocrine or craniofacial conditions were less likely to use dental care whereas children with hematologic or digestive conditions were more likely to use dental care. Children with respiratory, musculoskeletal, or ear/nose/throat conditions were more likely to use most types of dental care compared to other children with chronic conditions but without these specific conditions (RR: 1.03 to 1.13; 1.0 to 1.08; 1.02 to 1.12; respectively). There was no difference in use across all types of dental care for children with diabetes or cardiovascular conditions compared to other children with chronic conditions who did not have these particular conditions.

**Conclusions:**

Dental utilization is not homogeneous across chronic condition subgroups. Nearly 42% of children in our study did not use any dental care in 2006. These findings support the development of multilevel clinical interventions that target subgroups of Medicaid-enrolled children with chronic conditions that are most likely to have problems accessing dental care.

## Background

The 2011 Institute of Medicine Report *Improving Access to Oral Health Care for Vulnerable and Underserved Populations* highlights the problems children with chronic conditions have in accessing dental care [1]. Over 20% of children in the U.S. have chronic conditions [2,3]. Based on the definition of children with special health care needs developed by the Maternal and Child Health Bureau, chronic conditions are behavioral, intellectual, developmental, or physical ailments expected to last ≥12 months in ≥75% of patients identified with the condition [4]. Examples of common chronic conditions include uncontrolled asthma, attention deficit and hyperactivity disorder, and cerebral palsy.

Dental caries is the most common disease among all children, including those with chronic conditions [2,5]. As a group, children with chronic conditions are believed to be at increased risk for caries for the following reasons: (1) use of sugar-containing, acidic, or xerostomic medications; (2) frequent exposure to carbohydrates because of dietary needs or oromuscular problems; (3) behavioral co-morbidities that make it difficult for caregivers to brush the child’s teeth regularly with fluoridated toothpaste; and (4) dentists’ unwillingness to treat children with chronic conditions. A comprehensive strategy to ensure optimal oral health for children with chronic conditions includes regular visits to a dentist for preventive care (e.g., examinations; cleanings; topical fluoride; sealants) as well as restorative care (e.g., fillings; stainless steel crowns; extractions) when needed. However, dental care is the most common unmet health care need among children with chronic conditions [2], which has renewed interests in developing strategies aimed at improving dental utilization for medically vulnerable children.

Medicaid is the largest public source of dental care funding for children with chronic conditions in the U.S. [6]. State Medicaid programs are required by the federal Early and Periodic Screening, Diagnosis, and Treatment (EPSDT) Program to provide all child enrollees with comprehensive dental care [7]. While Medicaid-enrolled children are more likely to visit a dentist than uninsured children [8,9], studies have documented disparities in dental care use among subgroups of Medicaid-enrolled children [10,11]. A recent publication reported that Medicaid-enrolled children with chronic conditions are slightly more likely to use dental care than Medicaid-enrolled children without chronic conditions [12]. Compared to Medicaid-enrolled children with less complex chronic conditions, those with more complex chronic conditions were less likely to use any dental care [12]. In another study, Medicaid-enrolled children with an intellectual or developmental disability (defined as children with a non-acquired cognitive impairment) were equally as likely to use preventive dental care as Medicaid-enrolled children without an intellectual or developmental disability [13]. Collectively, these studies suggest heterogeneity in dental care use across subgroups of Medicaid-enrolled children with chronic conditions. While this is consistent with anecdotal evidence, there are no empirical studies to support this statement. The lack of data demonstrating heterogeneity in dental use may be one reason why current interventions fail to target children with chronic condition at greatest risk for disparities in dental use. Population-based interventions that target all children with chronic conditions are inefficient and may misallocate scare resources, which can lead to suboptimal outcomes.

In this study, we used 3M Clinical Risk Grouping (CRG) Software, a validated risk adjustment tool [14], to identify Medicaid-enrolled children with chronic conditions. Our goal was to assess dental use across 10 body system-based chronic condition subgroups. This approach is consistent with the specialty-focused medical care system into which most children with chronic conditions are integrated. Based on previous findings that children with chronic conditions have higher levels of unmet dental needs than those without [2], we compared dental care use for children with chronic conditions across each of the following chronic condition subgroups: hematologic; cardiovascular; craniofacial; diabetes; endocrine; digestive; ear/nose/throat; respiratory; catastrophic neurological; and musculoskeletal. The knowledge generated from this study will help us to identify the subgroups of children with chronic conditions who are at greatest risk for disparities in dental care use and to develop future interventions aimed at ensuring that these children have optimal access to dental care.

## Methods

### Study design

This was a retrospective study based on enrollment and claims data from the Iowa Medicaid Program (2003-2006). We received approvals from the University of Washington and the University of Iowa Institutional Review Boards.

### Conceptual model

The study was based on a sociocultural oral health disparities model presented by Patrick and colleagues [15]. Model covariates were organized into five domains:

· Ascribed factors (immutable individual-level determinants);

· Proximal factors (modifiable individual-level health behaviors);

· Immediate factors (household-level mediators between proximal and intermediate factors);

· Intermediate factors (community-level factors); and

· Distal factors (system-level factors).

### Study subjects

We focused on children with chronic conditions ages 3-14 years who were enrolled in the Iowa Medicaid Program for ≥11 months in 2005 and in 2006. Children under age 3 were excluded because chronic conditions are typically not diagnosed until the child’s third birthday [16]. We also excluded children ≥15 years of age because the determinants of dental use for older adolescents are different from younger children [17].

**Figure 1:**
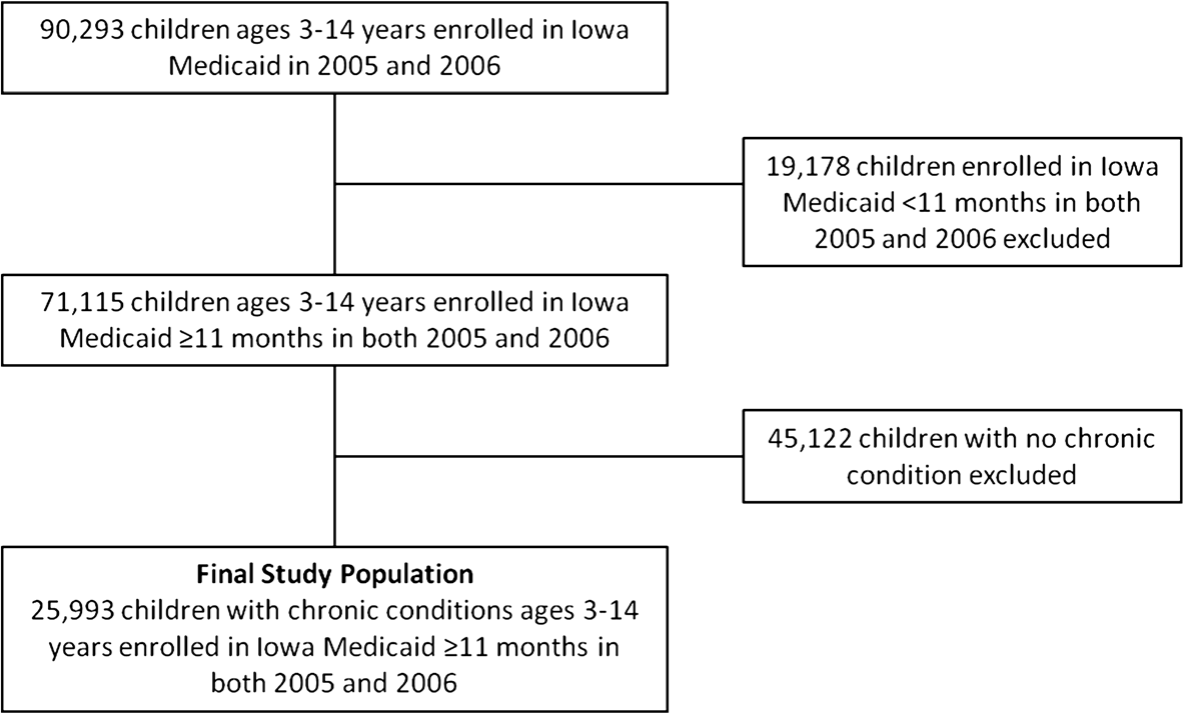
Flowchart on how final study population was derived.

Children with chronic conditions were identified by applying the 3M Clinical Risk Grouping (CRG) Software to each child’s medical claims data from 2003-2005 (Wallingford, CT) [18]. The CRG algorithm uses diagnostic codes (International Classification of Disease–Version Nine–Clinical Modification [ICD-9-CM]) and health service utilization codes (Current Procedural Terminology [CPT]) to classify each child into one of nine mutually exclusive Core Health Status Groups (CHSGs) [4]. We excluded children in CHSGs 1 (healthy children) or 2 (children with an acute condition) and focused on children in CHSGs 3 (minor chronic condition) through 9 (catastrophic chronic condition). The final study population consisted of 25,993 Iowa Medicaid-enrolled children with chronic conditions ages 3-14 years (Figure 1).

### Main exposure variables

The CRG Software also uses ICD-9-CM codes to classify each child into non-mutually exclusive body system-based Medical Diagnostic Categories (MDC). The diagnoses under each MDC correspond to a single organ system and are aligned with the delivery of specialty pediatric medical care [19]. We selected 10 MDCs most relevant for oral health: hematologic (MDC-161); cardiovascular (MDC-51); craniofacial (MDC-32); diabetes (MDC-101); digestive (MDC-61); endocrine (MDC-102); ear/nose/throat (MDC-31); respiratory (MDC-41); catastrophic neurological (MDC-12); and musculoskeletal (MDC-81). Each child with a chronic condition was classified as “no” or “yes” to indicate whether they were classified into each of the 10 MDCs.

### Outcome measure

The primary outcome measure was use of any dental care in 2006 (no/yes) [20]. Dental services were identified from encounter files using Current Dental Terminology (CDT) codes, which are five-digit alphanumeric codes used for billing. Secondary outcome measures included use of diagnostic care (e.g., examinations; D0110-D0330), preventive care (e.g., cleanings; topical fluoride treatment; sealants; D1110-D1351; D4355), routine restorative care (e.g., fillings; D2110-D2394), or complex restorative care (e.g., pulp therapy; stainless steel crowns; extraction; D1510-D1550; D2930-4342; D7110-D7140; D9420).

### Model covariates

Based on Patrick’s model [15], we considered the following 10 variables (organized into five domains) for inclusion in our models, measured in 2005.

Ascribed factors: age (three categories based on the child’s dentition: 3-7 [primary and early mixed dentition]; 8-12 [mixed dentition]; 13-14 [early permanent dentition] years); sex (male/female); race/ethnicity (White, Black, other, missing/unknown); and chronic condition severity (using previously validated methods [14], the seven CHSGs were reorganized into a four-category hierarchical, mutually exclusive variable referred to as modified CHSGs: episodic chronic condition; life-long chronic condition; malignancy; or catastrophic chronic condition).

Proximal factors: use of preventive medical care in 2005 (no/yes); previous use of any dental care in 2005 (no/yes).

Immediate factors: whether the child had any Medicaid-enrolled siblings (no/yes) or adults in the household (no/yes).

Intermediate factor: rurality (a four-category variable [13] based on the 2003 USDA Rural-Urban Continuum Codes and the child’s county of residence: metropolitan; urban adjacent to metropolitan; urban non-adjacent to metropolitan; rural).

Distal factor: whether the child lived in a dental Health Professional Shortage Area based on the child’s residential zip code (no/yes).

### Statistical analyses

After generating descriptive statistics, we used the Pearson chi-square test (α = 0.05) to test the bivariate relationships between model covariates and (1) the 10 chronic condition subgroups and (2) the five outcome measures. Next, we assessed for collinearity between the rurality and dental HPSA variables. There was no evidence of collinearity and both variables were included in the models. Then we constructed five Poisson regression models (use of any dental care, diagnostic care, preventive care, routine restorative care, and complex restorative care) for each of the 10 chronic condition subgroups. We reported covariate-adjusted relative risk ratios and estimated 95% confidence intervals using robust general estimating equation estimators of variance [21]. We tested for a statistical interaction between the two immediate factors (whether the child had a Medicaid-enrolled sibling or adult in the household) and included the interaction term in the regression model only if it was statistically significant. To address the problem of high correlation between use of any dental care in 2005 and the outcome measures, we dropped this variable from the final regression models. We analyzed the data using SPSS Version 19.0 for Windows.

## Results

### Descriptive statistics

The mean age for children in the study was 8.9 ± 3.4 years (data not shown). About 40% of children were female (Table 1). Over 70% were White; 9.1% were Black; 7.5% were another race or ethnicity; and 13.2% had missing/unknown race or ethnicity data. In regards to chronic condition severity 69.3% had episodic chronic conditions; 28.2% had life-long chronic conditions; 0.3% had a malignancy; and 2.2% had catastrophic chronic conditions. Nearly 90% of children utilized preventive medical care in 2005. About 67.1% had a Medicaid-enrolled sibling and 55.5% had an adult in their household enrolled in Medicaid. Most children lived in a metropolitan area (55.2%) and 65.8% lived in a dental HPSA.

**Table 1:** Medicaid-enrolled children (N = 25,993) by chronic condition subgroup

	**All children with a chronic condition ** **N = 25,993**	**Respiratory condition subgroup**		**Ear/nose/throat condition subgroup**		**Digestive condition subgroup**		**Musculoskeletal condition subgroup**		**Endocrine condition subgroup**	
		No	Yes	No	Yes	No	Yes	No	Yes	No	Yes
		n = 4,415	n = 21,578	n = 6,965	n = 19,028	n = 14,759	n = 11,234	n = 14,757	n = 11,236	n = 20,429	n = 5,564
		(17.0)	(83.0)	(26.8)	(73.2)	(56.8)	(43.2)	(56.8)	(43.2)	(78.6)	(21.4)
Ascribed factors											
Age (years)											
3-7	9,644 (37.1)	732 (16.6)	8,912 (41.3)*	413 (5.9)	9,231 (48.5)*	4,655 (31.5)	4,989 (44.4)*	6,060 (41.1)	3,584 (31.9)*	7,075 (34.6)	2,569 (46.2)*
8-12	11,321 (43.6)	2,452 (55.5)	8,869 (41.1)	4,305 (61.8)	7,016 (36.9)	6,982 (47.2)	4,339 (38.6)	6,542 (44.3)	4,779 (42.5)	9.248 (45.3)	2,073 (37.3)
13-14	5,028 (19.3)	1,231 (27.9)	3,797 (17.6)	2,247 (32.3)	2,781 (14.6)	3,122 (21.2)	1,906 (17.0)	2,155 (14.6)	2,873 (25.6)	4,106 (20.1)	922 (16.6)
Sex											
Female	10,434 (40.1)	1,461 (33.1)	8,973 (41.6)*	2,502 (35.9)	7,932 (41.7)*	5,604 (38.0)	4,830 (43.0)*	5,627 (38.1)	4,807 (42.8)*	8,102 (39.7)	2,332 (41.9) †
Race/ethnicity											
White	18,255 (70.2)	2,998 (67.9)	15,257 (70.7)*	4,892 (70.2)	13,363 (70.2)*	10,278 (69.6)	7,977 (71.0)*	9,916 (67.2)	8,336 (74.2)*	14,373 (70.4)	3,882 (69.8)*
Black	2,372 (9.1)	428 (9.7)	1,944 (9.0)	769 (11.0)	1,603 (8.4)	1,438 (9.7)	934 (8.3)	1,543 (10.5)	829 (7.4)	1,944 (9.5)	428 (7.7)
Other	1,944 (7.5)	263 (6.0)	1,681 (7.8)	411 (5.9)	1,533 (8.1)	1,058 (7.2)	886 (7.9)	1,203 (8.2)	741 (6.6)	1,529 (7.5)	415 (7.5)
Missing/Unknown	3,422 (13.2)	726 (16.4)	2,696 (12.5)	893 (12.8)	2,529 (13.3)	1,985 (13.4)	1,437 (12.8)	2,092 (14.2)	1,330 (11.8)	2,583 (12.6)	839 (15.1)
Chronic condition severity											
Episodic	18,025 (69.3)	2,936 (66.5)	15,089 (69.9)*	4,784 (68.7)	13,241 (69.6)	10,891 (73.8)	7,134 (63.5)*	10,706 (72.5)	7,319 (65.1)*	15,231 (74.6)	2,794 (50.2)*
Life-Long	7,324 (28.2)	1,352 (30.6)	5,972 (27.7)	2,020 (29.0)	5,304 (27.9)	3,672 (24.9)	3,652 (32.5)	3,881 (26.3)	3,443 (30.6)	4,896 (24.0)	2,428 (43.6)
Malignancy	85 (0.3)	18 (0.4)	67 (0.3)	16 (0.2)	69 (0.4)	27 (0.2)	58 (0.5)	41 (0.3)	44 (0.4)	36 (0.2)	49 (0.9)
Catastrophic	559 (2.2)	109 (2.5)	450 (2.1)	145 (2.1)	414 (2.2)	169 (1.1)	390 (3.5)	129 (0.9)	430 (3.8)	266 (1.3)	293 (5.3)
Proximal factors											
Child used preventive medical care in 2005	23,080 (88.8)	3,317 (75.1)	19,763 (91.6)*	5,562 (79.9)	17,518 (92.1)*	12,583 (85.3)	10,497 (93.4)*	12,722 (86.2)	10,358 (92.2)*	17,875 (87.5)	5,205 (93.5)*
Child used any dental care in 2005	15,398 (59.2)	2,456 (55.6)	12,942 (60.0)*	4,039 (58.0)	11,359 (59.7) ‡	8,605 (58.3)	6,793 (60.5)*	8,449 (57.3)	6,949 (61.8)*	12,217 (59.8)	3,181 (57.2)*
Immediate factors											
Child had at least one Medicaid- enrolled sibling	17,449 (67.1)	2,799 (63.4)	14,650 (67.9)*	4,625 (66.4)	12,824 (67.4)	10,287 (69.7)	7,162 (63.8)*	10,077 (68.3)	7,372 (65.6)*	14,286 (69.9)	3,163 (56.8)*
Child had at least one Medicaid- enrolled adult	14,436 (55.5)	1,841 (41.7)	12,595 (58.4)*	3,377 (48.5)	11,059 (58.1)*	7,879 (53.4)	6,557 (58.4)*	7,999 (54.2)	6,437 (57.3)*	11,604 (56.8)	2,832 (50.9)*
Immediate factor											
Rurality											
Metropolitan	14,353 (55.2)	2,653 (60.1)	11,700 (54.2)*	4,071 (58.4)	10,282 (54.0)*	8,261 (56.0)	6,092 (54.2) ‡	8,391 (56.9)	5,962 (53.1)*	11,327 (55.4)	3,026 (54.4)
Urban adjacent to metropolitan	4,978 (19.2)	812 (18.4)	4,166 (19.3)	1,304 (18.7)	3,674 (19.3)	2,765 (18.7)	2,213 (19.7)	2,716 (18.4)	2,262 (20.1)	3,883 (19.0)	1,095 (19.7)
Urban non-adjacent to metropolitan	5,237 (20.1)	730 (16.5)	4,507 (20.9)	1,239 (17.8)	3,998 (21.0)	2,917 (19.8)	2,320 (20.7)	2,870 (19.4)	2,367 (21.1)	4,091 (20.0)	1,146 (20.6)
Rural	1,425 (5.5)	220 (5.0)	1,205 (5.6)	351 (5.0)	1,074 (5.6)	816 (5.5)	609 (5.4)	780 (5.3)	645 (5.7)	1,128 (5.5)	297 (5.3)
Distal factor											
Child lived in a dental Health Professional Shortage Area	17,110 (65.8)	2,876 (65.1)	14,234 (66.0)	4,616 (66.3)	12,494 (65.7)	9,753 (66.1)	7,357 (65.5)	9,798 (66.4)	7,312 (65.1) ‡	13,555 (66.4)	3,555 (63.9) †

Pearson Chi Square test to evaluate bivariate relationship between model covariates and each chronic condition.

* p < .0001.

† p < .01.

‡ p < .05.

The proportions of children in the non-mutually exclusive chronic condition subgroups, in descending order, are as follows: respiratory (83.0%); ear/nose/throat (73.2%); digestive (43.2%); musculoskeletal (43.2%); endocrine (21.4%); cardiovascular (10.2%); hematologic (6.4%); catastrophic neurological (2.5%); craniofacial (1.8%); and diabetes (1.4%) (Tables 1 and 2).

**Table 2:** Medicaid-enrolled children (N = 25,993) by chronic condition subgroup

	**Cardiovascular condition subgroup**		**Hematologic condition subgroup**		**Catastrophic neurological condition subgroup**		**Craniofacial condition subgroup**		**Diabetes subgroup**	
	No	Yes	No	Yes	No	Yes	No	Yes	No	Yes
	n = 23,340	n = 2,653	n = 24,325	n = 1,668	n = 25,356	n = 637	n = 25,517	n = 476	n = 25,642	n = 351
	(89.8)	(10.2)	(93.6)	(6.4)	(97.5)	(2.5)	(98.2)	(1.8)	(98.6)	(1.4)
Ascribed factors										
Age (years)										
3-7	8,651 (37.1)	993 (37.4)*	8,719 (35.8)	925 (55.5)*	9,388 (37.0)	256 (40.2)	9,383 (36.8)	261 (54.8)*	9.545 (37.2)	99 (28.2)*
8-12	10,262 (44.0)	1,059 (39.9)	10,829 (44.5)	492 (29.5)	11,069 (43.7)	252 (39.6)	11,172 (43.8)	149 (31.3)	11,174 (43.6)	147 (41.9)
13-14	4,427 (19.0)	601 (22.7)	4,777 (19.6)	251 (15.0)	4,899 (19.3)	129 (20.3)	4,962 (19.4)	66 (13.9)	4,923 (19.2)	105 (29.9)
Sex										
Female	9,353 (40.1)	1,081 (40.7)	9,757 (40.1)	677 (40.6)	10,158 (40.1)	276 (43.3)	10,250 (40.2)	184 (38.7)	10,264 (40.0)	170 (48.4)‡
Race/ethnicity										
White	16,393 (70.2)	1,862 (70.2)	17,191 (70.7)	1,064 (63.8)*	17,790 (70.2)	465 (73.0)*	17,928 (70.3)	327 (68.7)*	18,002 (70.2)	253 (72.1)
Black	2,133 (9.1)	239 (9.0)	2,181 (9.0)	191 (11.5)	2,356 (9.3)	16 (2.5)	2,347 (9.2)	25 (5.3)	2,338 (9.1)	34 (9.7)
Other	1,724 (7.4)	220 (8.3)	1,783 (7.3)	161 (9.7)	1,913 (7.5)	31 (4.9)	1,913 (7.5)	31 (6.5)	1,924 (7.5)	20 (5.7)
Missing/Unknown	3,090 (13.2)	332 (12.5)	3,170 (13.0)	252 (15.1)	3,297 (13.0)	125 (19.6)	3,329 (13.0)	93 (19.5)	3,378 (13.2)	44 (12.5)
Chronic condition severity										
Episodic	16,720 (71.6)	1,305 (49.2)*	17,052 (70.1)	973 (58.3)*	17,981 (70.9)	44 (6.9)*	17,810 (69.8)	215 (45.2)*	17,957 (70.0)	68 (19.4)*
Life-Long	6,124 (26.2)	1,200 (45.2)	6,728 (27.7)	596 (35.7)	7,180 (28.3)	144 (22.6)	7,082 (27.8)	242 (50.8)	7,066 (27.6)	258 (73.5)
Malignancy	58 (0.2)	27 (1.0)	48 (0.2)	37 (2.2)	84 (0.3)	1 (0.2)	85 (0.3)	0 (0.0)	83 (0.3)	2 (0.6)
Catastrophic	438 (1.9)	121 (4.6)	497 (2.0)	62 (3.7)	111 (0.4)	448 (70.3)	540 (2.1)	19 (4.0)	536 (2.1)	23 (6.6)
Proximal factors										
Child used preventive medical care in 2005	20,602 (88.3)	2,478 (93.4)*	21,480 (88.3)	1,600 (95.9)*	22,471 (88.6)	609 (95.6)*	22,631 (88.7)	449 (94.3)*	22,751 (88.7)	329 (93.7)‡
Immediate factors										
Child had at least one Medicaid-enrolled sibling	15,857 (67.9)	1,592 (60.0)*	16,401 (67.4)	1,048 (62.8)*	17,326 (68.3)	123 (19.3)*	17,200 (67.4)	249 (52.3)*	17,214 (67.1)	235 (67.0)
Child had at least one Medicaid-enrolled adult	13,016 (55.8)	1,420 (53.5) ‡	13,457 (55.3)	979 (58.7)†	14,330 (56.5)	106 (16.6)*	14,228 (55.8)	208 (43.7)*	14,235 (55.5)	201 (57.3)
Immediate factor										
Rurality										
Metropolitan	12,871 (55.1)	1,482 (55.9)	13,431 (55.2)	922 (55.3)	13,962 (55.1)	391 (61.4)‡	14,095 (55.2)	258 (54.2)	14,170 (55.3)	183 (52.1)
Urban adjacent to metropolitan	4,486 (19.2)	492 (18.5)	4,673 (19.2)	305 (18.3)	4,873 (19.2)	105 (16.5)	4,891 (19.2)	87 (18.3)	4,898 (19.1)	80 (22.8)
Urban non-adjacent to metropolitan	4,707 (20.2)	530 (20.0)	4,901 (20.1)	336 (20.1)	5,122 (20.2)	115 (18.1)	5,134 (20.1)	103 (21.6)	5,171 (20.2)	66 (18.8)
Rural	1,276 (5.5)	149 (5.6)	1,320 (5.4)	105 (6.3)	1,399 (5.5)	26 (4.1)	1,397 (5.5)	28 (5.9)	1,403 (5.5)	22 (6.3)
Distal factor										
Child lived in a dental Health Professional Shortage Area	15,351 (65.8)	1,759 (66.3)	16,023 (65.9)	1,087 (65.2)	16,768 (66.1)	342 (53.7)*	16,809 (65.9)	301 (63.2)	16,879 (65.8)	231 (65.8)

Pearson Chi Square test to evaluate bivariate relationship between model covariates and each chronic condition.

* p < .0001.

† p < .01.

‡ p < .05.

### Bivariate statistics

The bivariate relationships between model covariates and exposure variables (each of the 10 chronic condition subgroups) are summarized in Tables 1 and 2. Even though most children were male, when there were statistically significant differences, larger proportions of children across the chronic condition subgroups were female. Across every subgroup, significantly larger proportions of children with the chronic condition utilized preventive medical care in 2005 than children without the specific chronic condition. There were no other consistent findings.

The bivariate relationships between model covariates and the primary outcome variable (any dental care use in 2006) as well as the secondary outcome measures (use of diagnostic, preventive, routine restorative, or complex restorative dental care) are summarized in Table 3. Significantly larger proportions of children who utilized each type of dental care were White. In addition, significantly larger proportions of children who utilized preventive medical care in 2005 subsequently utilized all types of dental care in 2006.

**Table 3:** Relationships between covariates and dental utilization outcome measures

	**Any dental care use in 2006**		**Diagnostic dental care use in 2006**		**Preventive dental care use in 2006**		**Routine restorative dental care use in 2006**		**Complex restorative dental care use in 2006**	
	No	Yes	No	Yes	No	Yes	No	Yes	No	Yes
	n = 10,846 (41.7)	n = 15,147 (58.3)	n = 11,772 (45.3)	n = 14,221 (54.7)	n = 13,119 (50.5)	n = 12,874 (49.5)	n = 21,116 (81.2)	n = 4,877 (18.8)	n = 23,637 (90.9)	n = 2,356 (9.1)
Main predictor variables (chronic condition subgroups)										
Respiratory condition										
No	2,017 (18.6)	2,398 (15.8)*	2,227 (18.9)	2,188 (15.4)*	2,427 (18.5)	1,988 (15.4)*	3,656 (17.3)	759 (15.6)†	4,078 (17.3)	337 (14.3)*
Yes	8,829 (81.4)	12,749 (84.2)	9,545 (81.1)	12,033 (84.6)	10,692 (81.5)	10,886 (84.6)	17,460 (82.7)	4,118 (84.4)	19,559 (82.7)	2,019 (85.7)
Ear/nose/throat chronic condition										
No	3,025 (27.9)	3,940 (26.0)‡	3,336 (28.3)	3,629 (25.5)*	3,751 (28.6)	3,214 (25.0)*	5,650 (26.8)	1,315 (27.0)	6,433 (27.2)	532 (22.6)*
Yes	7,821 (72.1)	11,207 (74.0)	8,436 (71.7)	10,592 (74.5)	9,368 (71.4)	9,660 (75.0)	15,466 (73.2)	3,562 (73.0)	17,204 (72.8)	1,824 (77.4)
Digestive condition										
No	6,286 (58.0)	8,473 (55.9)†	6,842 (58.1)	7,917 (55.7)*	7,555 (57.6)	7,204 (56.0)‡	11,937 (56.5)	2,822 (57.9)	13,469 (57.0)	1,290 (54.8)‡
Yes	4,560 (42.0)	6,674 (44.1)	4,930 (41.9)	6,304 (44.3)	5,564 (42.4)	5,670 (44.0)	9,179 (43.5)	2,055 (42.1)	10,168 (43.0)	1,066 (45.2)
Musculoskeletal condition										
No	6,373 (58.8)	8,384 (55.4)*	6,891 (58.5)	7,866 (55.3)*	7,532 (57.4)	7,225 (56.1)‡	12,102 (57.3)	2,655 (54.4)*	13,438 (56.9)	1,319 (56.0)
Yes	4,473 (41.2)	6,763 (44.6)	4,881 (41.5)	6,355 (44.7)	5,587 (42.6)	5,649 (43.9)	9,014 (42.7)	2,222 (45.6)	10,199 (43.1)	1,037 (44.0)
Endocrine condition										
No	8,508 (78.4)	11,921 (78.7)	9,249 (78.6)	11,180 (78.6)	10,272 (78.3)	10,157 (78.9)	16,408 (77.7)	4,021 (82.4)*	18,559 (78.5)	1,870 (79.4)
Yes	2,338 (21.6)	3,226 (21.3)	2,523 (21.4)	3,041 (21.4)	2,847 (21.7)	2,717 (21.1)	4,708 (22.3)	856 (17.6)	5,078 (21.5)	486 (20.6)
Cardiovascular condition										
No	9,743 (89.8)	13,597 (89.8)	10,586 (89.9)	12,754 (89.7)	11,747 (89.5)	11,593 (90.0)	18,938 (89.7)	4,402 (90.3)	21,242 (89.9)	2,098 (89.0)
Yes	1,103 (10.2)	1,550 (10.2)	1,186 (10.1)	1,467 (10.3)	1,372 (10.5)	1,281 (10.0)	2,178 (10.3)	475 (9.7)	2,395 (10.1)	258 (11.0)
Hematologic condition										
No	10,182 (93.9)	14,143 (93.4)	11,047 (93.8)	13,278 (93.4)	12,307 (93.8)	12,018 (93.4)	19,746 (93.5)	4,579 (93.9)	22,101 (93.5)	2,224 (94.4)
Yes	664 (6.1)	1,004 (6.6)	725 (6.2)	943 (6.6)	812 (6.2)	856 (6.6)	1,370 (6.5)	298 (6.1)	1,536 (6.5)	132 (5.6)
Catastrophic neurological condition										
No	10,491 (96.7)	14,865 (98.1)*	11,386 (96.7)	13,970 (98.2)*	12,721 (97.0)	12,635 (98.1)*	20,527 (97.2)	4,829 (99.0)*	23,054 (97.5)	2,302 (97.7)
Yes	355 (3.3)	282 (1.9)	386 (3.3)	251 (1.8)	398 (3.0)	239 (1.9)	589 (2.8)	48 (1.0)	583 (2.5)	54 (2.3)
Craniofacial condition										
No	10,648 (98.2)	14, 865 (98.1)*	11,386 (96.7)	13,964 (98.2)	12,864 (98.1)	12,653 (98.3)	20,703 (98.0)	4,814 (98.7)‡	23,198 (98.1)	2,319 (98.4)
Yes	198 (1.8)	282 (1.9)	386 (3.3)	257 (1.8)	255 (1.9)	221 (1.7)	413 (2.0)	63 (1.3)	439 (1.9)	37 (1.6)
Diabetes										
No	10,690 (98.6)	14,952 (98.7)	11,603 (98.6)	14,039 (98.7)	12,933 (98.6)	12,709 (98.7)	20,822 (98.6)	4,820 (98.8)	23,207 (98.6)	2,335 (99.1)‡
Yes	156 (1.4)	195 (1.3)	169 (1.4)	182 (1.3)	186 (1.4)	165 (1.3)	294 (1.4)	57 (1.2)	330 (1.4)	21 (0.9)
Ascribed factors										
Age (years)										
3–7	4,023 (37.1)	5,621 (37.1)	4,287 (36.4)	5,357 (37.7)*	4,588 (35.0)	5,056 (39.3)*	7,969 (37.7)	1,675 (34.3)*	8,659 (36.6)	985 (41.8)*
8–12	4,60 2(42.4)	6,719 (44.4)	5,042 (42.8)	6,279 (44.2)	5,424 (41.3)	5,897 (45.8)	9,208 (43.8)	2,113 (43.3)	10,218 (43.2)	1.103(46.8)
13–14	2,221 (20.5)	2,807(18.5)	2,443 (20.8)	2,585 (18.12)	3,107 (23.7)	1,921 (14.9)	3,939 (18.7)	1,089 (22.3)	4,760 (20.1)	268 (11.4)
Sex										
Female	4,178 (38.5)	6,256 (41.3)*	4,544 (38.6)	5,890 (41.4)*	5,147 (39.2)	5,287(41.1)^	8,436 (40.0)	1,998 (41.0)	9,483 (40 1)	951 (40.4)
Race/ethnicity										
White	7,400(68.2)	10.855 (71.7)*	8,069 (68.5)	10,186 (71.6)*	9,099 (69.4)	9,156 (71.1)	14,696(69.8)	3,559 (73.0)*	16,539 (70.0)	1.716 (72.8)*
Black	1,140 (10.5)	1,232 (8.1)	1,193 (10.1)	1,179 (8.3)	1,303 (9.9)	1,069 (8.3)	1,992 (9.4)	380 (7.8)	2,220 (9.4)	152 (6.5)
Other	817 (7.5)	1,127 (7.4)	883 (7.5)	1,061 (7.5)*	948(72)	996 (7.7)	1,562(7.4)	382 (7.8)	1,751 (7.4)	193 (8.2)
Missing Unknown	1,489 (13.7)	1,933 (12.8)	1,627 (13.8)	1,795 (12.6)	1,653 (13.5)	1,653 (12.8)	2,866 (13.6)	556 (11.4)	3,127 (13.2)	295 (12.5)
Chronic condition severity										
Episodic	7,504 (69.2)	10,521 (69.5)*	8,139 (69.1)	9,886 (69.5)*	9,037 (68.9)	8,988 (69.8)*	14,488 (68.8)	3,537 (72.5)	16,396 (69.4)	1,629 (69.1)
Life-Long	3,019 (27.8)	4,305 (28.4)	3,279 (27.9)	4,045 (28.4)	3,705 (28.2)	3,619 (28.1)	1,992 (9.4)	1,269 (26.0)	6,654 (28.2)	670 (28.4)
Malignancy	33 (0.3)	52 (0.3)	37 (0.3)	48 (0.3)	43 (0.3)	42 (0.3)	1,562 (7.4)	20 (0.4)	72 (0.3)	13 (0.6)
Catastrophic	290 (2.7)	269 (1.8)	317 (2.7)	242 (1.7)	334 (2.5)	225 (1.7)	2,866 (13.6)	51 (1.0)	515 (2.2)	44 (1.9)
Proximal factors										
Child used preventive medical care in 2005	9,391 (86.6)	13,689 (90.4)*	10,205 (86.7)	12,875 (90.5)*	11,437 (87.2)	11,643 (90.4)*	18,691 (88.5)	4,389 (90.0)†	20,969 (88.7)	2,111 (89.6)
Immediate factors										
Child had at least one Medicaid- enrolled sibling	7,200 (66.4)	10,249 (67.7)‡	7,816 (66.4)	9,633 (67.7)‡	8,677 (66.1)	8,772 (68.1)†	13,971 (66.2)	3,478 (71.3)*	15,806 (66.9)	1,643 (69.7)†
Child had at least one Medicaid- enrolled adult	6,097 (56.2)	8,339 (55.1)	6,528 (55.5)	7,908 (55.6)	7,334 (55.9)	7,102 (55.2)	11,595 (54.9)	2,841 (58.3)*	13,036 (55.2)	1,400 (59.4)*
Immediate factor										
Rurality										
Metropolitan	5,854 (54.0)	8,499 (56.1)†	6,363 (54.1)	7,990 (56.2)†	6,958 (53.0)	7,395 (57.4)*	11,631 (55.1)	2,722 (55.8)	13,007 (55.0)	1,346 (57.1)
Urban adjacent to metropolitan	2,123 (19.6)	2,855 (18.8)	2,308 (19.6)	2,670 (18.8)	2,607 (19.9)	2,371 (18.4)	4,088 (19.4)	890 (18.2)	4,545 (19.2)	433 (18.4)
Urban non-adjacent to metropolitan	2,275 (21.0)	2,962 (19.6)	2,456 (20.9)	2,781 (19.6)	2,802 (21.4)	2,435 (18.9)	4,264 (20.2)	973 (20.0)	4,783 (20.2)	454 (19.3)
Rural	594 (5.5)	831 (5.5)	645 (5.5)	780 (5.5)	752 (5.7)	673 (5.2)	1,133 (5.4)	292 (6.0)	1,302 (5.5)	123 (5.2)
Distal factor										
Child lived in a dental Health Professional Shortage Area	7,260 (66.9)	9,850 (65.0)†	7,821 (66.4)	9,289 (65.3)	8,718 (66.5)	8,392 (65.2)‡	13,898 (65.8)	3,215 (65.9)	15,566 (65.9)	1,544 (65.5)

Pearson Chi Square test to evaluate bivariate relationship between model covariates and each outcome measure.

* p < .0001.

† p < .01.

‡ p < .05.

### Unadjusted dental utilization in 2006

About 58.3% of Medicaid-enrolled children with chronic conditions used any dental care in 2006; 54.7% used diagnostic care; 49.5% used preventive care; 18.8% used routine restorative care; and 9.1% used complex restorative care (Table 4).

**Table 4:** Dental utilization rates by chronic condition subgroups and across covariates

**All children with a chronic condition**										
**N = 25,993**										
	**Utilized any dental care in 2006**		**Utilized any diagnostic dental care in 2006**		**Utilized any preventive dental care in 2006**		**Utilized any routine restorative dental care in 2006**		**Utilized any complex restorative dental care in 2006**	
	n = 15,147 (58.3)		n = 14,221 (54.7)		n = 12,874 (49.6)		n = 4,877 (18.8)		n = 2,356 (9.1)	
	n	%	n	%	n	%	n	%	n	%
Main predictor variables (chronic condition subgroups)										
Respiratory condition										
No	2,398	54.3*	2,188	49.6*	1,988	45.0*	759	17.2‡	337	7.6*
Yes	12,749	59.1	12,033	55.8	10,886	50.4	4,118	19.1	2,019	9.4
Ear/nose/throat chronic condition										
No	3,940	56.6‡	3,629	52.1*	3,214	46.1*	1,315	18.9	532	7.6*
Yes	11,207	58.9	10,592	55.7	9,660	50.8	3,562	18.7	1,824	9.6
Digestive condition										
No	8,473	57.4‡	7,917	53.6*	7,204	48.8‡	2,822	19.1	1,290	8.7‡
Yes	6,674	59.4	6,304	56.1	5,670	50.5	2,055	18.3	1,066	9.5
Musculoskeletal condition										
No	8,384	56.8*	7,866	53.3*	7,225	49.0‡	2,655	18.0*	1,319	8.9
Yes	6,763	60.2	6,355	56.6	5,649	50.3	2,222	19.8	1,037	9.2
Endocrine condition										
No	11,921	58.4	11,180	54.7	10,157	49.7	4,021	19.7*	1,870	9.2
Yes	3,226	58.0	3,041	54.7	2,717	48.8	856	15.4	486	8.7
Cardiovascular condition										
No	13,597	58.3	12,754	54.6	11,593	49.7	4,402	18.9	2,098	9.0
Yes	1,550	58.4	1,467	55.3	1,281	48.3	475	17.9	258	9.7
Hematologic condition										
No	14,143	58.1	13,278	54.6	12,018	49.4	4,579	18.8	2,224	9.1
Yes	1,004	60.1	943	56.5	856	51.3	298	17.9	132	7.9
Catastrophic neurological condition										
No	14,865	58.6*	13,970	55.1*	12,635	49.8*	4,829	19.0*	2,303	9.1
Yes	282	44.3	251	39.4	239	37.5	48	7.5	54	8.5
Cranofacial condition										
No	14,869	58.3	13,964	54.7	12,635	49.6	4,814	18.9‡	2,319	9.1
Yes	278	58.4	257	54.0	239	46.4	63	13.2	37	7.8
Diabetes										
No	14,952	58.3	14,039	54.8	12,709	49.6	4,820	18.8	2,335	9.1‡
Yes	195	55.6	182	51.9	165	47.0	57	16.2	21	
Ascribed factors										
Age (years)										
3–7	5,621	58.3*	5,357	55.5*	5,056	52.4*	1,675	17.4*	985	10.2*
8–12	6,719	59.3	6,279	55.5	5,897	52.1	2,113	18.7	1,103	9.7
13–14	2,807	55.8	2,585	51.4	1,921	38.2	1,089	21	268	5.3
Sex										
Female	6,256	60.0*	5,890	56.5*	5,287	50.7‡	1,998	19.1	951	9.1
Male	8,891	57.1	8,331	53.5	7,587	48.8	2,879	18.5	1,405	9.0
Race/ethnicity										
White	10,855	59.5*	10,186	55.8*	9,156	50.2*	3,559	19.5*	1,716	9.4*
Black	1,232	51.9	1,179	49.7	1,069	45.1	380	16.0	152	6.4
Other	1,127	58.0	1,061	54.6	996	51.2	382	19.7	193	9.9
Missing/Unknown	1,933	56.5	1,795	52.5	1,653	48.3	556	16.2	295	8.6
Chronic condition severity										
Episodic	10,521	58.4*	9,886	54.8*	8,988	49.9*	3,537	19.6*	1,629	9.0
Life-Long	4,305	58.8	4,045	55.2	3,619	49.4	1,269	17.3	670	9.1
Malignancy	52	61.2	48	56.5	42	49.4	20	23.5	13	15.3
Catastrophic	269	48.1	242	43.3	225	40.3	51	9.1	44	7.9
Proximal factors										
Child used preventive medical care in 2005										
No	1,458	50.1*	1,346	46.2*	1,231	43.3*	488	16.8†	245	8.4
Yes	13,689	59.3	12,875	55.8	11,643	50.4	4,389	19.0	2,111	9.1
Immediate factors										
Child had at least one Medicaid-enrolled sibling										
No	4,898	57.3‡	4,588	53.7‡	4,102	48.0‡	1,399	16.4*	713	8.3†
Yes	10,249	58.7	9,633	55.2	8,772	50.3	3,478	19.9	1,643	9.4
Child had at least one Medicaid-enrolled adult										
No	6,808	58.9	6,313	54.6	5,772	49.9	2,036	17.6*	956	8.3*
Yes	8,339	57.8	7,908	54.8	7,102	49.2	2,841	19.7	1,400	9.7
Immediate factor										
Rurality										
Metropolitan	8,499	59.2†	7,990	55.7†	7,395	51.5*	2,722	19.0	1,346	9.4
Urban adjacent to metropolitan	2,855	57.4	2,670	53.6	2,371	47.6	890	17.9	433	8.7
Urban non-adjacent to metropolitan	2,962	56.6	2,781	53.1	2,435	46.5	973	18.6	454	8.7
Rural	831	58.3	780	54.7	673	47.2	292	20.5	123	8.6
Distal factor										
Child lived in a dental Health Professional Shortage Area										
No	5,297	59.6†	4,932	55.5	4,482	50.5‡	1,662	18.7	812	9.1
Yes	9,850	57.6	9,289	54.3	8,392	49.0	3,215	18.8	1,544	9.0

Pearson Chi Square test to evaluate bivariate relationship between each chronic condition and outcome measure.

* p < .0001.

† p < .01.

‡ p < .05.

Significantly lower proportions of children with catastrophic neurological conditions used all types of dental care, expect for complex restorative care, for which there was no difference. Larger proportions of children with respiratory, ear/nose/throat, digestive, or musculoskeletal conditions used most types of dental care than did children with chronic conditions without these specific conditions. There was no difference in use across all types of dental care for children with and without diabetes or cardiovascular conditions. Utilization was inconsistent across the different types of dental care for children with hematologic, endocrine, or craniofacial conditions and those children with chronic conditions but without these specific conditions.

In regards to other variables, significantly larger proportions of children ages 3-7 and 8-12 years utilized all types of dental care, except routine restorative care, than did children ages 13-14 years. Compared to children with the least severe chronic conditions (episodic), larger proportions of children with a malignancy (the second highest severity group) utilized all types of dental care except for preventive dental care whereas children with a catastrophic condition (the most severe chronic condition group) utilized all types of dental care at the lowest rates.

Larger proportions of children who utilized preventive medical care in 2005 subsequently utilized dental care in 2006. Significantly larger proportions of children with a Medicaid-enrolled sibling utilized all types of dental care, whereas this relationship was statistically significant only for routine and complex restorative dental care for the children with a Medicaid-enrolled adult in the household. Significantly larger proportions of children in metropolitan areas utilized any, preventive, or diagnostic dental care; there were no significant differences by rurality for routine and complex restorative dental care. Finally, children who lived in a dental HPSA were less likely to utilize all types of dental care, though these differences were significant only for any dental care and for preventive dental care.

### Poisson regression models

The statistical interaction between the two immediate factors, having a Medicaid-enrolled sibling or adult in the household, was statistically significant for routine restorative dental care use across all 10 chronic condition subgroups and for preventive dental care use for some of the chronic condition subgroups. The interaction term was included only in the models in which it was statistically significant. Covariate-adjusted relative risks (RR) corresponding to the 10 chronic condition subgroups are summarized in Table 5. Relative risks for other model covariates are available upon request. Our findings are organized into 4 groupings.

**Table 5:** Covariate-adjusted relative risk (RR) associated with dental use across chronic condition subgroups

	**Models A**						**Model B**		**Model C**	
	**Utilized any dental care in 2006**		**Utilized any diagnostic dental care in 2006**		**Utilized any preventive dental care in 2006**		**Utilized any routine restorative dental care in 2006**		**Utilized any complex restorative dental care in 2006**	
	RR*	95% CI	RR	95% CI	RR*	95% CI	RR†	95% CI	RR*	95% CI
Main exposure variables (reference group = no)										
Respiratory condition	1.06	1.03, 1.10	1.09*	1.05, 1.13	1.07	1.03, 1.11	1.10	1.02, 1.19	1.13	1.01, 1.26
Ear/nose/throat condition	1.02	0.99, 1.05	1.04†	1.01, 1.07	1.03	1.01, 1.07	1.03	0.97, 1.10	1.12	1.01, 1.24
Digestive condition	1.03	1.01, 1.05	1.03†	1.01, 1.06	1.02	0.99, 1.04	0.98	0.93, 1.04	1.04	0.96, 1.13
Musculoskeletal condition	1.06	1.04, 1.08	1.06†	1.04, 1.09	1.06	1.03, 1.08	1.08	1.02, 1.13	1.08	0.99, 1.17
Endocrine condition	0.99	0.96, 1.02	0.99†	0.97, 1.02	0.97	0.94, 1.01	0.83	0.77, 0.89	0.92	0.84, 1.02
Cardiovascular condition	1.00	0.97, 1.04	1.01†	0.97, 1.04	0.98	0.94, 1.02	0.98	0.90, 1.06	1.09	0.96, 1.23
Hematologic condition	1.04	0.99, 1.08	1.03†	0.99, 1.08	1.03	0.98, 1.08	0.99	0.89, 1.10	0.82	0.70, 0.98
Catastrophic neurological condition	0.72	0.63, 0.81	0.68*	0.60, 0.78	0.73	0.63, 0.84	0.48	0.34, 0.67	1.11	0.79, 1.56
Craniofacial condition	0.99	0.92, 1.07	0.98†	0.90, 1.06	0.92	0.83, 1.01	0.76	0.61, 0.96	0.83	0.61, 1.13
Diabetes	0.95	0.87, 1.05	0.95†	0.86, 1.05	0.98	0.87, 1.10	0.89	0.71, 1.13	0.67	0.44, 1.02

* Models adjusted for age, sex, race/ethnicity, chronic condition severity, preventive medical care use in 2005, Medicaid-enrolled sibling, Medicaid-enrolled adult, rurality, and living in a dental Health Professional Shortage Area.

† Models adjusted for age, sex, race/ethnicity, chronic condition severity, preventive medical care use in 2005, Medicaid-enrolled sibling, Medicaid-enrolled adult, statistical interaction between Medicaid-enrolled sibling and Medicaid-enrolled adult, rurality, and living in a dental Health Professional Shortage Area.

First, children with catastrophic neurological conditions were significantly less likely (RR: 0.48 to 0.73) to use most types of dental care than other children with chronic conditions but without a catastrophic neurological condition. There was no difference in complex restorative dental care use (p = .56). Children with an endocrine condition were slightly less likely to use preventive care and routine restorative dental care than children with chronic conditions but without an endocrine condition (p = .049 and p < .0001; respectively). Children with craniofacial conditions were also less likely to use routine restorative care and children with hematologic conditions were less likely to utilize complex restorative dental care than children with chronic conditions who did not have these particular conditions (p = .02 and p = .03; respectively). In other words, when there were differences, children with catastrophic neurological, endocrine, craniofacial, or hematologic conditions were less likely to utilize dental care than children with chronic conditions but without these specific conditions.

Second, children with respiratory or musculoskeletal conditions were significantly more likely to use most types of dental care than other children with chronic conditions but without these specific conditions (RR: 1.06 to 1.13 and 1.06 to 1.08; respectively). Among children with chronic conditions, there was no difference in complex restorative dental care use for children with and without musculoskeletal conditions. Children with ear/nose/throat conditions were significantly more likely to use diagnostic, preventive, and complex restorative dental care and there was no difference in use of any or routine restorative dental care. Children with digestive conditions were significantly more likely to use any dental care or diagnostic dental care than other children with chronic conditions without these specific conditions (RR: 1.03 for both types of dental care). There was no difference in use of the other three types of dental care. In other words, when there were differences, children with respiratory, musculoskeletal, ear/nose/throat, or digestive conditions were significantly were more likely to utilize dental care than other children with chronic conditions who did not have these particular chronic conditions.

Third, there was no significant difference across all five outcome measures for children with diabetes or cardiovascular conditions and children with other types of chronic conditions but without these specific conditions.

Fourth, in regards to other model covariates there are three sets of findings (data not shown). In the any, diagnostic, and preventive dental care use models (Models A), children in the following subgroups were significantly less likely to use dental care: children ages 13-14 (referent = ages 3-7); males; Blacks (referent = Whites); children with the most severe chronic health conditions; children who did not use preventive medical care in 2005; children without a Medicaid-enrolled sibling; those living in urban areas (referent = metropolitan); and those living in a dental HPSA. In the routine restorative dental care use models (Model B), findings were similar to those from Models A except that children ages 13-14 were more likely to use routine restorative care. There were no significant differences in the risk ratios of routine restorative dental care use across sex and dental HPSA status. For the complex restorative dental care use models (Model C), findings were similar to those from Models A except that there were no significant differences across sex, whether the child used preventive medical care in 2005, whether the child had a Medicaid-enrolled sibling, or whether the child lived in a dental HPSA.

## Discussion

This is the first known study, to our knowledge, that examined dental care use for Medicaid-enrolled children with chronic conditions with an emphasis on body system-based subgroups. We compared dental care use for Medicaid-enrolled children across 10 chronic condition subgroups. Collectively, our data support two findings that are new to the dental health services literature: (1) dental care use is heterogeneous across chronic condition subgroups; and (2) the determinants of dental care use vary across different types of dental care.

There were three main findings in regards to specific chronic conditions. The first is that when there were differences children in certain subgroups (e.g., catastrophic neurologic, endocrine, craniofacial, hematologic conditions) were significantly less likely to use dental care than other children with chronic conditions who did not have these particular conditions. Children with these chronic conditions may be at the greatest risk for disparities in dental care use. There are two possible explanations. Many of these children have developmental or acquired cognitive deficits and may have difficulty cooperating during dental visits. Dentists could be less willing to treat these children because of inadequate training [22]. Another explanation is that caregivers may have high levels of stress associated with managing the child’s other systemic health care needs [23], which pushes oral health down on the priority list. It is particularly worrisome that children with catastrophic neurologic conditions were significantly less likely to use preventive dental care. This finding has oral health-related implications especially if the child has a poor diet or behavioral comorbidities that make it difficult for caregivers to brush the child’s teeth regularly with fluoridated toothpaste. These findings appear to conflict with previous work suggesting that Medicaid-enrolled children with intellectual or developmental disabilities are equally as likely to use preventive dental care as those without [13]. A possible explanation for this inconsistency is that children with intellectual or developmental disabilities present with varying degrees of disability. The previous study did not control for this factor while the current study did.

The second finding is that children with respiratory, musculoskeletal, ear/nose/throat, or digestive conditions were more likely to use most types of dental care compared to children with other types of chronic conditions but without these spe-cific conditions. Children with respiratory conditions (e.g., asthma, cystic fibrosis) may require medications or have enamel defects – factors that increase their risk for dental caries [24-26]. Children with musculoskeletal conditions (e.g., arthritis) are also at risk for oral health problems [27]. Children with ear/nose/throat conditions undergo procedures involving the mouth and oral structures, making it plausible that these children receive team-based medical care. These factors may increase caregiver awareness of the importance of dental visits or the likelihood of dental referrals by physicians, though there are no published data to support these hypotheses. Studies from the medical literature report low adherence to inhaler medication for Medicaid-enrolled children with asthma because of caregiver misunderstanding of medications, which makes the former explanation unlikely [28]. We recognize that the risk ratios from our models are small (ranging from 1.02 to 1.13). However, on a population-level, small risk ratios are meaningful, especially when the prevalence of a particular chronic condition is high [29]. The prevalence of respiratory conditions was over 80% and over 40% of children in our study had a musculoskeletal or ear/nose/throat condition. Identifying the mechanisms underlying higher rates of dental use for children with specific types of chronic conditions in future studies may provide insight on how to improve utilization rates for children in other chronic condition subgroups that are not as likely to use dental care.

The third finding is that there was no difference in dental use for children with diabetes or cardiovascular conditions compared to children with other chronic conditions but without these conditions. Non-significant differences in dental care use may not be a clinically significant problem as long as children are receiving appropriate dental care. However, this is unlikely, especially because these chronic conditions have oral health-related sequelae that make dental visits important. For instance, the link between pediatric diabetes and periodontal disease [30,31] underscores the importance of regular maintenance and monitoring therapy that might require additional dental visits for children with diabetes. Future studies should investigate whether no differences in dental care use across subgroups actually means that children in these subgroups are receiving appropriate dental care.

In addition to the findings related to specific chronic conditions, we found that children who used preventive medical care are significantly more likely to use all types of dental care, except for complex restorative care. While there is potential for selection bias [32], this finding reinforces the importance of strengthening the clinical ties between pediatric medicine and dentistry [33]. The mechanisms between use of medical and dental care have not yet been elucidated and require further investigation.

In term of the research significance of the our study, any dental care use, a standard measure of access to dental care services, may be a more appropriate proxy for use of diagnostic or preventive dental care services rather than routine or complex restorative dental care. When developing oral health intervention and polices, it may be most effective for planners to specify the particular types of dental care the program seeks to improve use of by taking into consideration the differential determinants of dental care. This maximizes the likelihood that children have appropriate access to preventive as well as restorative dental care when needed [34].

As with all studies, our investigation has strengths and limitations. The primary strength is that we used validated methods, 3M Clinical Risk Groups, to identify children with chronic conditions and to adjust for the severity of those chronic conditions in the models. In addition, we adopted an a priori conceptual model that helped to guide model covariate selection. Finally, we examined use of different types of dental care to obtain a more complete view of dental utilization for children with chronic conditions. The major limitation is the lack of clinical oral health data, which precluded us from determining whether the observed utilization rates were appropriate. This limitation can be addressed with future studies by collecting clinical data and linking these data with dental claims data. Another limitation is that we measured dental use during a single calendar year, which provides a snapshot rather than a longitudinal perspective on dental use. Future studies might examine utilization trends over time across the different chronic condition subgroups. Finally, because this was an observational study, there is potential for residual confounding, which we attempted to minimize by adopting a conceptual model that we used to develop our empirical model. In the future, there is the potential to link claims data with survey data that might be used to collect social and behavioral measures that potentially confound the relationship between chronic conditions and dental use.

## Conclusion

The goal of pediatric dentistry is to ensure optimal oral health for all children, including children with chronic conditions. An important component of optimal oral health is regular visits to the dentist for preventive dental care and restorative care when needed. Our findings suggest heterogeneous dental utilization patterns for children across different chronic condition subgroups. It is important to note that nearly 42% of children in our study did not utilize any dental care in 2006, which highlights the barriers to dental care that many Medicaid-enrolled children with chronic condition encounter. Some of these barriers may be system-level (e.g., low reimbursement to dentists for treatment) whereas others are environmental/social (e.g., lack of dental offices in areas where Medicaid enrollees live) or behavioral (e.g., dentists’ unwillingness to see Medicaid patients or symptom-driven dental utilization patterns by patients). The next step for researchers is to identify the social and behavioral determinants of particular types of dental care use that exist at these various levels (e.g., ascribed, proximal, immediate, intermediate, distal). This information can then be used to develop and test multilevel clinical interventions aimed at improving dental utilization for specific subgroups of children with chronic conditions who exhibit the greatest disparities in dental care use.

## Competing interests

The authors declare that they have no competing interests.

## Authors’ contributions

DC conceived of the study, designed the study, conducted the statistical analyses, and drafted the manuscript. NR conducted the statistical analyses and drafted the manuscript. All authors read and approved the final manuscript.

## Pre-publication history

The pre-publication history for this paper can be accessed here:

http://www.biomedcentral.com/1472-6831/12/28/prepub
